# Rapid and Accurate Identification by Real-Time PCR of Biotoxin-Producing Dinoflagellates from the Family Gymnodiniaceae

**DOI:** 10.3390/md12031361

**Published:** 2014-03-07

**Authors:** Kirsty F. Smith, Miguel de Salas, Janet Adamson, Lesley L. Rhodes

**Affiliations:** 1 Cawthron Institute, 98 Halifax Street East, Private Bag 2, Nelson 7042, New Zealand; E-Mails: janet.adamson@cawthron.org.nz (J.A.); lesley.rhodes@cawthron.org.nz (L.L.R.); 2 Tasmanian Herbarium, Tasmanian Museum and Art Gallery, Private Bag 4, Hobart, Tasmania 7001, Australia; E-Mail: dr.microbe@gmail.com

**Keywords:** real-time PCR, large subunit ribosomal RNA (LSU rRNA) gene, dinoflagellate monitoring, biotoxin production

## Abstract

The identification of toxin-producing dinoflagellates for monitoring programmes and bio-compound discovery requires considerable taxonomic expertise. It can also be difficult to morphologically differentiate toxic and non-toxic species or strains. Various molecular methods have been used for dinoflagellate identification and detection, and this study describes the development of eight real-time polymerase chain reaction (PCR) assays targeting the large subunit ribosomal RNA (LSU rRNA) gene of species from the genera *Gymnodinium*, *Karenia*, *Karlodinium*, and *Takayama.* Assays proved to be highly specific and sensitive, and the assay for *G. catenatum* was further developed for quantification in response to a bloom in Manukau Harbour, New Zealand. The assay estimated cell densities from environmental samples as low as 0.07 cells per PCR reaction, which equated to three cells per litre. This assay not only enabled conclusive species identification but also detected the presence of cells below the limit of detection for light microscopy. This study demonstrates the usefulness of real-time PCR as a sensitive and rapid molecular technique for the detection and quantification of micro-algae from environmental samples.

## 1. Introduction

The dinoflagellate family Gymnodiniaceae includes the genera *Gymnodinium*, *Karenia*, *Karlodinium*, and *Takayama* [1,2]. Species from the genera *Gymnodinium* and *Karenia* have been responsible for fish kills and shellfish contamination events worldwide [3,4] including New Zealand [5,6,7,8]. The only toxic *Gymnodinium* species, *G. catenatum* was first recorded along the northwest coastline of New Zealand following the detection of paralytic shellfish poisons (PSP) in shellfish in May 2000. During that bloom event, PSP toxicity reached 4027 μg saxitoxin equivalents/100 g in Greenshell™ mussels (*Perna canaliculus*) [7]. A number of species in the *Karenia* genus have been reported to cause severe blooms including *K. brevis*, *K. mikimotoi*, *K. brevisulcata*, *K. selliformis*, *K. longicanalis*, and *K. digitata* [6,9,10,11]. The first recorded major bloom of a *Karenia* species in New Zealand occurred in 1992/93 along the coast of Northland. At that time 180 cases of illness that fitted the symptoms of neurotoxic shellfish poisoning (NSP) were reported [5,12,13]. The identity of the causative organism was not definitely determined, but later confirmed as *K. mikimotoi* [13]. Brevetoxins and brevetoxin analogues were detected in shellfish samples causing the total closure of all bivalve industries in New Zealand [12]. In addition to brevetoxins, *Karenia* species are known to produce gymnodimines [11,14], *Karenia brevisulcata* toxins (KBTs) and brevisulcatic acids (BSXs) [15], the ichthyotoxic gymnocins A and B [16,17], and haemolytic glycolipids that cause gill damage in fish and have been linked to fish kills in Japan and Norway [11]. Additionally, species from the genera *Karlodinium* and *Takayama* have been implicated in fish kills worldwide [18].

Aside from their negative impacts on food safety, the biotoxins and compounds produced by Gymnodiniaceae species are of interest for commercial exploitation and potential medical applications [19]. For example, compounds (karlotoxins) produced by *Karlodinium veneficum* have been investigated for application as non-toxic cholesterol pharmacophores [20]. Due to their molecular complexity the main method for obtaining these compounds from dinoflagellates is still extraction and purification from laboratory cultures of cells isolated from environmental samples [21] and, in some cases, via contaminated shellfish tissue [22]. Because of the large variability in the type of compounds produced even within a species, accurate identification of biotoxin-producing species from both cultures and environmental samples is crucial.

Routine phytoplankton monitoring of seawater is carried out weekly at approximately 100 sites around New Zealand to inform shellfish harvesters of the potential for toxins in shellfish [23,24]. Analyses are currently carried out at the Cawthron Institute (Nelson, New Zealand), with results expected within 24 h. This monitoring data is critical for shellfish harvesting management decisions in New Zealand. Species in the genus *Karenia* can be difficult to distinguish from each other under the light microscope [11] and are identified as *Karenia* cf. *mikimotoi* for the New Zealand Non-Commercial Marine Biotoxin Monitoring Programme [25]. This term encompasses the following species: *K. mikimotoi*, *K. bidigitata*, *K. brevisulcata*, *K. papilionacea*, *K. selliformis*, *Karlodinium veneficum* and *Gymnodinium impudicum.* Efforts to differentiate *G. catenatum* from look-alike, non-toxic species, e.g., *G. impudicum*, using light microscopy can also be difficult and the morphology of *G. catenatum* cells are often variable [26].

The rapid and accurate identification of toxin-producing dinoflagellate species is essential to assess the risk of bloom formation that can negatively impact human health, marine ecosystems, and aquaculture activities [27,28,29,30], and to aid with isolation of the valuable bioactive compounds produced by these species [19]. Monitoring programmes typically involve microscopic examination of water samples, which requires considerable taxonomic experience [31]. Additionally, the species of interest may only occur as a minor component of the community and it can be difficult to morphologically differentiate between toxic and non-toxic species or even strains, e.g., the *Alexandrium tamarense* species complex [32]. In recent years the application of molecular methods for the detection of dinoflagellate species has increased, as these methods are generally rapid, species-specific and do not require specialised expertise [33]. Various molecular methods have been utilised for dinoflagellate detection each with its own advantages and disadvantages [31,33,34], including most commonly: fluorescence *in situ* hybridisation assay (FISH) [26,35,36], sandwich hybridisation assay (SHA) [26,37,38], and traditional or real-time polymerase chain reaction (PCR) [39,40,41,42,43,44,45,46,47]. Several types of real-time PCR assays with differing levels of specificity have been developed and positive reactions are detected either with a fluorescent reporter probe (e.g., hydrolysis probes, molecular beacons, locked-nucleic acid bases (LNA)) or a double-stranded DNA-binding dye (e.g., SYBR green). More recently, microarray technologies have demonstrated the ability to detect numerous species simultaneously [48,49].

This study describes the development and optimisation of eight real-time PCR assays, all targeting the large subunit ribosomal RNA (LSU rRNA) gene, for the detection of a range of toxic and morphologically similar non-toxic *Karenia*, *Gymnodinium*, *Karlodinium*, and *Takayama* species to assist with toxic dinoflagellate monitoring programmes [24] as well as chemical and ecological research.

## 2. Results and Discussion

In this study eight real-time PCR assays were developed for the dinoflagellate species *Gymnodinium aureolum*, *G. catenatum*, *Karenia brevisulcata*, *K. mikimotoi*, *K. papilionacea*, *K. umbella*, *Karlodinium veneficum*, and *Takayama tasmanica*, all targeting the D1-D3 region of LSU rRNA gene. Designed assays ranged from 93 to 232 bp in length (Table 1). All of the assays, except for the *G. aureolum* assay, amplified only the target species as determined via cross-reactivity testing with strains listed in Table 2. The *in silico* analysis using NCBI blast also showed that primers and probes did not match with other species. Positive results for the *G. aureolum* assay were obtained for the strains *Gymnodinium* sp. (CAWD71), *G. chlorophorum* (CAWD62) and *G.* cf. *microreticulatum* (CAWD191). However, the assay did not cross-react with *K. mikimotoi*. The assay was primarily developed to differentiate the non-toxic *G. aureolum* from the morphologically similar toxic *K. mikimotoi* and so is still useful for this purpose.

**Table 1:** Sequences of primers and probes designed in this study including optimised final concentrations for real-time PCR assays.

Target Species	Primer Name and Sequence	Product Size (bp)	Final Concentration
*Gymnodinium aureolum*	GA519-F: GGACATGGTAGCCTGCC	153	500 nM
	GA683-R: GTCAGGAAGGTGCTCAGC		500 nM
	GA560-P: 6FAM-CAGAACTCACTGTCATATTGCTCCTCC-BHQ-1		50 nM
*Gymnodinium catenatum*	GC397-F: CTTGGTGAGATTGTCGCAC	93	500 nM
	GC471-R: GCAAGAAACATCACACCGA		1000 nM
	GC426-P: 6FAM-TGATCACCTTCTATTCCAGCGAAAGC-BHQ-1		80 nM
*Karenia brevisulcata*	KBS460-F: GATCTGGATGCGATACTGAAT	153	300 nM
	KBS585-R: AGCACTGCTACAAGACATATAA		900 nM
	KBS544-P: 6FAM-TGACTGAATGTCCCTAGTTGAACTC-BHQ-1		50 nM
*Karenia mikimotoi*	KM541-F: CGAGTGACTGAATGTCCTCA	112	500 nM
	KM645-R: CCAACAACCTTCATGCAGAG		250 nM
	KM578-P: 6FAM-CTACCAGACACACAGAGAGCAG-BHQ-1		50 nM
*Karenia papilionacea*	KP449-F: TCTGGATGCGATACTGGTTG	232	1000 nM
	KP682-R: TACTTATGTCAAGGATGTGTTC		750 nM
	KP630-P: 6FAM-CTTGTTAGTTACCTGGCATGAGAC-BHQ-1		125 nM
*Karenia umbella*	KU480-F: ATGTCAACGTCAGTTCACAAT	161	750 nM
	KU623-R: GCACGAGACGAGGCTTA		250 nM
	KU542-P: 6FAM-TTCGACTAGGCACATTCAGTCAC-BHQ-1		50 nM
*Karlodinium veneficum*	KV590-F: TGCCTGGTAGAACTCATGTC	100	1000 nM
	KV672-R: ACGAGTAACAGAAGCTACAAG		1000 nM
	KDV640-P: 6FAM-TGTTCTCATTACCTGCGTCTGGG-BHQ-1		50 nM
*Takayama tasmanica*	TT533-F: ACTTCTGGGTGACTGAACGT	134	100 nM
	TT665-R: CCACGTCCTGTCCCATGC		1000 nM
	TT616-P: 6FAM-CTGGGCTTTGTTCACTGCTCTTAA-BHQ-1		125 nM

**Table 2:** The dinoflagellate strains with corresponding Cawthron Institute Culture Collection of Micro-algae (CICCM) codes used in this study. Accession numbers included are for sequences from the target species used to design the real-time PCR assays.

Species Name	CICCM Code	Accession Number
*Gymnodinium aureolum*	CAWD59, 87	AY947659
*Gymnodinium catenatum*	CAWD102, 101, 109, 126	AY036128
*Gymnodinium* cf. *impudicum*	CAWD139	
*Gymnodinium* cf. *microreticulatum*	CAWD191	
*Gymnodinium chlorophorum*	CAWD62	
*Gymnodinium impudicum*	CAWD03	
*Gymnodinium instriatum*	CAWD137	
*Gymnodinium simplex*	CAWD86	
*Gymnodinium* sp.	CAWD172	
*Karenia bidigitata*	CAWD80	
*Karenia brevis*	CAWD08	
*Karenia brevisulcata*	CAWD82	AY243032
*Karenia mikimotoi*	CAWD63, 117, 133, 134, 192	U92249
*Karenia papilionacea*	CAWD91	U92252
*Karenia selliformis*	CAWD79	
*Karenia umbella*	CAWD131, 65	AY947664
*Karlodinium veneficum*	CAWD84	AY947665
*Takayama helix*	CAWD128	
*Takayama tasmanica*	CAWD115	AY947669

**Figure 1:**
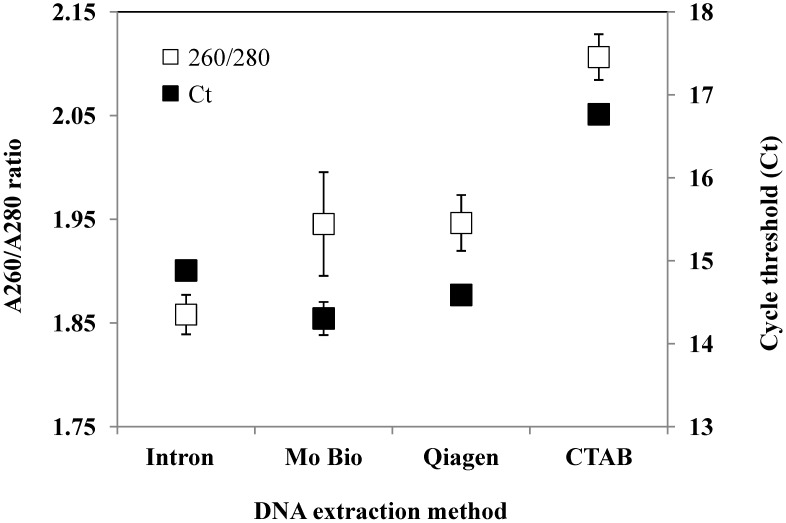
Mean A260/A280 ratios and cycle threshold (C*t*) values for replicate DNA extractions of *Karenia mikimotoi*. Error bars are ± standard error of three replicate DNA extractions.

The assays all had various optimised primer and probe concentrations (Table 1). The DNA extraction method that gave the best A260/A280 ratios and lowest Ct values was the PowerSoil^®^ DNA isolation kit (Mo Bio, Carlsbad, CA, USA) (Figure 1). The PowerSoil^®^ DNA isolation kit was also selected as these kits are optimised for the removal of environmental PCR inhibitors and environmental samples showed no evidence of PCR inhibition.

The real-time PCR assays had a linear range of detection six to eight orders of magnitude with a limit of detection (LOD) well below one cell for all assays (Table 3). This is similar to the LOD reported for other real-time PCR assays for Gymnodiniaceae species [50,51]. The amplification efficiency of all assays was between 93% and 106% (Table 3).

**Table 3:** Range of detection, amplification efficiency and *R*^2^ values.

Target Species	Lower Limit of Detection (Cells/Reaction, 1 s.f.)	Amplification Efficiency	*R*^2^ Value
*K. mikimotoi*	0.007	101%	1.00
*K. umbella*	0.09	105%	0.99
*K. papilionacea*	0.2	95%	0.99
*K. brevisulcata*	0.2	102%	0.99
*K. veneficum*	0.3	93%	0.98
*G. catenatum*	0.006	106%	1.00
*G. aureolum*	0.006	105%	0.99
*T. tasmanica*	0.09	102%	1.00

For the last two decades, species belonging to the family Gymnodiniaceae have caused a number of toxic events along the New Zealand coastline. Additionally, blooms of these species have caused major damage to marine ecosystems and aquaculture internationally during the past 60 years [15]. All assays developed in this study are regularly utilised by the micro-algae laboratory at the Cawthron Institute for confirmation of species identification during routine examination of samples as part of the New Zealand Marine Phytoplankton Monitoring Programme [24]. The identification of *Karenia* species by LM is particularly difficult and thus cells in field samples are often identified as *K.* cf. *mikimotoi*. The conclusive identification of a potentially toxic species is most difficult when cell concentrations are low or the species is rarely encountered in routine monitoring. For example, in 1998 the southern coast of the North Island of New Zealand experienced a severe HAB, which devastated almost all the marine life in Wellington Harbour [52]. A new *Karenia* species was isolated from the bloom and named *Karenia brevisulcata*. This species is similar in morphology to other *Karenia* species and has never been reported since. If this species were to bloom again it could have devastating impacts on marine ecosystems, aquaculture activities and human health. Additionally, the toxins produced by this and other *Karenia* species are novel bioactive compounds of great interest [15]. Accurate and early identification is vital and the assays designed in this study enable the conclusive identification of species from environmental samples with results available the same day as sample receipt. This is an important consideration for routine monitoring programmes that require a 24-h turn-around for results, but also for the mining of environmental samples for specific bioactive compound producers.

The copy number of the LSU rRNA gene per cell was determined for *G. catenatum*. The standard curve of serially diluted PCR product had a regression equation of *y* = −3.17 + 19.60*x*, *R*^2^ = 1.0 and an amplification efficiency of 107%. This is similar to the regression curve and amplification efficiency of the standard curve generated by cell number (Table 3). The mean copy number of the cultured strain was 20,800 ± 1566 copies per cell cell. This is comparable to values calculated for other dinoflagellate species [43,46,53]. The estimated LSU rRNA gene copy number calculated in this study also corresponds to the finding from Godhe *et al.* [54] that found the number of rDNA copies per cell is significantly correlated to the biovolume of dinoflagellate cells (*y* = −0.61 + 1.22*x*, *R*^2^ = 0.75; *x* = log rDNA molecules cell^−1^, *y* = log biovolume μm^3^ cell^−1^). The average biovolume of *G. catenatum* cells from the culture used to calculate copy number was 38,438 μm^3^, which equates to 23,335 copies of rDNA molecules cell^−1^ from the regression equation in Godhe *et al.* [54].

The *G. catenatum* assay was further developed for quantification to demonstrate the potential for the analysis of environmental samples and in response to the detection of naturally occurring cells in Manukau Harbour, New Zealand. The saxitoxin producing species *G. catenatum* was first detected in New Zealand in 2000 [7], although there is some evidence that this species may have been present in New Zealand prior to this, with a large range expansion in 2000 [8]. *Gymnodinium catenatum* has increased its geographic range around the entire North Island coastline of New Zealand over the last 12 years, and regular blooms are common in some areas (New Zealand Food Safety Authority, [55]). The ability to differentiate *G. catenatum* from morphologically similar, non-toxic species (e.g., *G. impudicum*) during routine monitoring using LM can be difficult, particularly at the onset of blooms when only a few individual cells per litre are present in seawater samples. Additionally, the morphology of *G. catenatum* cells can also be variable and be present as either single cells or chains [26].

**Figure 2:**
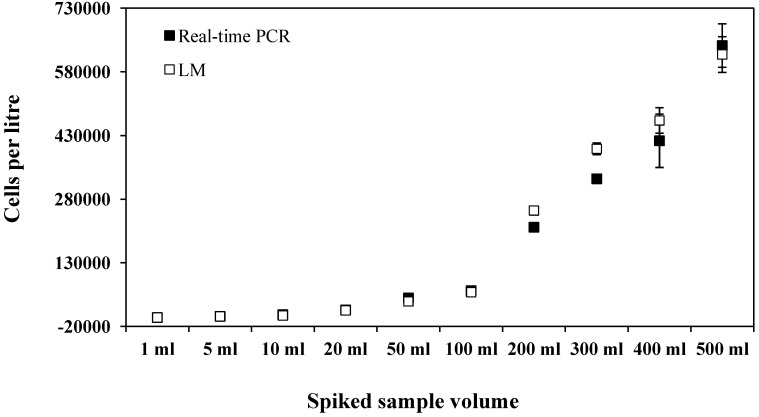
*Gymnodinium catenatum* cell number estimates by real-time PCR and light microscopy (LM) from natural phytoplankton samples spiked with cultured *G. catenatum* cells. Error bars are ±standard error from triplicate LM analyses and real-time PCR assays.

**Figure 3:**
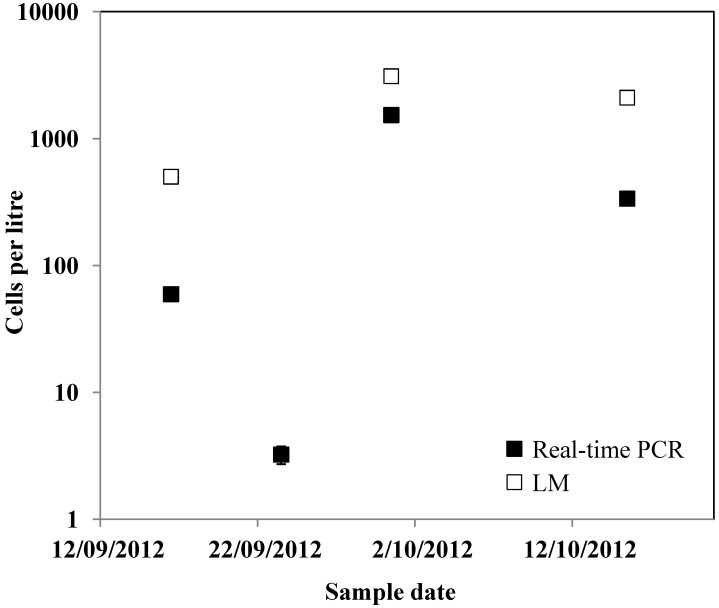
*Gymnodinium catenatum* cell number estimates by real-time PCR and light microscopy (LM) from samples collected at Manukau Bay, New Zealand. Error bars are ±standard error from replicate real-time PCR assays.

Estimates of *G. catenatum* cells per litre determined by the real-time PCR assay were generally similar to or slightly fewer than LM estimates, except at low levels below the limit of detection for LM (Figure 2 and Figure 3). Real-time PCR cell estimates ranged between 70% and 108% of LM cell estimates. This is comparable to what has been found for other real-time PCR assays [50] and has been proposed to be due to factors such as DNA recovery, PCR inhibitors and the exponential nature of PCR [46]. To test for PCR inhibition, the DNA extracts were diluted 1:10 and re-amplified. This did not alter the results and the assay successfully detected cell numbers ranging from below 10 cells per reaction to over 5000 cells per reaction in the spiked samples, which equated to 700 to over 600,000 cells per litre. For natural samples the assay detected a range of cell estimates from 0.07 to 16 cells per reaction, which equated to 3 to 1528 cells per litre. The sample size analysed by the two methods was very different (*i.e.*, 10 mL *versus* 300 mL) and as *G. catenatum* can occur as both single cells and chains of variable length (more than 60 cells/chain) [7] reliable subsampling can be difficult. LM analysis did not detect *G. catenatum* from samples collected in Manukau Harbour on the 23 September 2012, but the real-time PCR assay detected approximately three cells per litre (Figure 3). All positive environmental samples from Manukau Harbour were sequenced and sequences were identical. Using blastn searches, the highest homology found was with *G. catenatum* (e.g., GenBank acc. no. AY036128, coordinates 395-487: query coverage = 100%, E-value = 5 × 10^−15^, percent identity = 100%). Due to the difficulties in thoroughly testing all assays for specificity, we recommend DNA sequencing to confirm species identification especially during the initial stages of assay development.

The *G. catenatum* quantitative real-time PCR assay not only enabled conclusive identification but also detected the presence of cells at the LOD for LM. In this study the analysis of samples by LM and Utermöhl chambers has a lower LOD of 100 cells per litre [31]. This is at the threshold for triggering toxin testing in shellfish. As the real-time PCR assay has a LOD of less than one cell, *G. catenatum* could be reliably detected in field samples that were negative using LM. This lower LOD allows far greater forewarning of potentially toxic blooms for shellfish harvesters.

## 3. Experimental Section

### 3.1. Culture Maintenance

Dinoflagellate cultures (Table 2) were maintained in GP, 50% GP [56], or F/2 [57] medium at 100 µmol photons m^−2^ s^−1^ (14:10 h light:dark), 19 °C (±1 °C). The Cawthron Institute Culture Collection of Micro-algae (CICCM) provided the strains used in this study (Table 2). Cultures were harvested during exponential growth phase for assay optimisation or cross-reactivity testing.

### 3.2. DNA Extraction

Exponentially growing cultures of *Karenia mikimotoi* (CAWD63, CAWD117, CAWD133, CAWD134, CAWD192) were pooled and twelve 30 mL subsamples were filtered (Durapore membrane filters, 0.45 µm, Millipore, Billerica, MA, USA) and frozen overnight (−20 °C). DNA extraction was assessed using four methods (three replicates of each); (i-genomic CTB DNA extraction mini kits (Intron, Gyeonggi-do, South Korea), PowerSoil^®^ DNA isolation kit (Mo Bio, Carlsbad, CA, USA), DNeasy mini plant kit (Qiagen, Alameda, CA, USA), and a cetyltrimethylammonium bromide (CTAB) method [58]. All DNA extractions were eluted into 50 μL and quantified using a NanoPhotometer (Implen, Munich, Germany) to check for DNA quantity and quality (A260/A280 ratio). Each DNA extraction was assessed using 10 ng of *K. mikimotoi* genomic DNA with the real-time PCR conditions described above.

### 3.3. Primers and Probe Design for Real-Time PCR Assays

The target positions for forward and reverse primers and the hydrolysis probes were designed using a multiple LSU rRNA gene (D1-D3 region) alignments (ClustalW) [59] of the target species and sequences from closely related species obtained from GenBank. Separate assays were designed for the detection of eight species including *Gymnodinium aureolum*, *G. catenatum*, *Karenia brevisulcata*, *K. mikimotoi*, *K. papilionacea*, *K. umbella*, *Karlodinium veneficum*, and *Takayama tasmanica*. The specificity of the primer sequences was then confirmed using BLAST (Basic Local Alignment Search Tool) at NCBI (National Centre for Biotechnology Information). The hydrolysis probes were synthesized (GeneWorks, Adelaide, Australia) with 6-FAM reporter dye at the 5′-end and Black Hole Quencher 1 at the 3′-end (Table 1).

### 3.4. Real-Time PCR Assay Optimisation, Specificity and Sensitivity

Real-time PCR assays were optimised on a Rotor-Gene 6000 (Corbett, Sydney, NSW, Australia), using genomic DNA extracted from an exponentially growing culture of the target species. The optimised assays consisted of a 20 μL reaction containing 10 μL of Platinum^®^ Quantitative PCR SuperMix-UDG (Invitrogen, Carlsbad, CA, USA), 0.8 μg non-acetylated bovine serum albumin (BSA; Sigma-Aldrich, Auckland, New Zealand), and 10 ng of DNA template. Optimised primer and probe concentrations for each assay are shown in Table 1. All PCR reactions in this study were set up manually and all included no template control reactions. Assays were run in clear 0.2 mL thin-wall PCR tubes (Axygen, Union City, CA, USA). All assays had an optimised annealing temperature of 60 °C and PCR cycling conditions were: 50 °C for 2 min, 95 °C for 2 min and 45 cycles of 95 °C for 15 s and 60 °C for 45 s.

The specificity of each assay was verified using DNA from closely related species (Table 2). DNA from each species (10 ng) was used in the real-time PCR assays as described above. The sensitivity of each assay was evaluated with genomic DNA extracted using PowerSoil^®^ DNA isolation kits from known cell concentrations of the target species. The amplification efficiency of the assay was determined by using serially diluted genomic DNA samples (analysed in duplicate) ranging from approximately 100,000 to 1 × 10^−4^ cells per reaction and the corresponding cycle threshold (C*t*) data.

### 3.5. Determination of Copy Number and Quantification of the *Gymnodinium catenatum* Assay

The assay specific for *G. catenatum* was further developed in order to demonstrate the potential for quantification of field samples. DNA was extracted from replicate samples of known numbers of cells of the CAWD126 strain of *G. catenatum*. Cell concentrations of culture were estimated during exponential growth phase using the Utermöhl technique [60]. Replicates samples consisting of 150,000 cells were were filtered (Durapore membrane filters, 0.45 µm, Millipore, Billerica, MA, USA), frozen overnight (−20 °C) and transferred to the first tube of a PowerSoil^®^ DNA isolation kit. These extractions were serially diluted and used to generate a standard curve of known cell number per reaction *versus* Ct data.

To estimate the LSU rRNA gene copy number per cell a dilution series of a known concentration of LSU rRNA gene PCR product, ranging from 1 to 0.001 ng was analysed together with extractions of known cell number from above. The number of molecules of PCR product was then determined by the formula: (A × 6.022 × 10^23^) × (660 × B)^−1^, with A being the concentration of the PCR product and B the length of the PCR product. The number of molecules in the extractions with known cell number was determined using the PCR product standard curve to obtain the copy number of the LSU rRNA gene per cell. The average biovolume of cells from the culture used was also calculated to determine the relationship between cell size and gene copy number following Godhe *et al.* [54].

### 3.6. Spiked Environmental and Natural Sample Testing

The effectiveness of the real-time PCR assay for the identification and discrimination of *G. catenatum* from phytoplankton samples was examined using samples spiked with cultured cells. A surface seawater sample (10 L) was collected from Nelson Marina (Nelson, South Island, New Zealand). Different volumes (1, 5, 10, 20, 50, 100, 200, 300, 400 and 500 mL) of *G. catenatum* culture (mix of CAWD101, CAWD102, CAWD109 and CAWD126) were made up to one litre volume with the natural phytoplankton sample to create ten contrived samples. From the one litre samples triplicate 10 mL aliquots were preserved in Lugol’s solution analysed by Light Microscopy (LM) and triplicate 300 mL aliquots were analysed using the real-time PCR assay. For real-time PCR analyses samples were filtered and genomic DNA extracted with PowerSoil^®^ DNA isolation kits (Mo Bio, Carlsbad, CA, USA) as described above. To estimate the abundance of *G. catenatum* in environmental samples C*t* values were used to calculate cell number per reaction based on the standard curve generated with the serial dilution of DNA extracts of known cell number (ranging from 6000 to 0.006 cells per reaction). The real-time PCR assays all included standard curves, positive controls, negative controls and blank extraction controls. DNA samples were also diluted 1:10 to determine evidence of PCR inhibition.

Samples were collected from Manukau Harbour (Auckland, North Island, New Zealand) as part of the New Zealand Marine Phytoplankton Monitoring Programme (by the New Zealand Food Safety Authority, Ministry for Primary Industries, Wellington, New Zealand). Unpreserved and preserved (Lugol’s solution [60]) replicate samples were received within 24 h of collection. Grab samples (100 mL) from three depths (0, 3 and 6 m) were pooled (total 300 mL). *G. catenatum* cells were identified by LM and cell number estimates estimated using the Utermöhl technique [61] by the micro-algae laboratory at the Cawthron Institute, New Zealand (International Accreditation New Zealand: ISO 17025). One 10 mL subsample, from the pooled 300 mL, was analysed by LM for each environmental sample. For real-time PCR analysis the unpreserved grab samples filtered and genomic DNA extracted with PowerSoil^®^ DNA isolation kits (Mo Bio, Carlsbad, CA, USA) as described above. The abundance of *G. catenatum* in environmental samples was calculated as above. Environmental samples were also PCR amplified for DNA sequencing (Sanger sequencing) to confirm positive results. PCR amplifications were carried out in 50 μL reaction volumes containing i-Taq 26 PCR master mix (25 μL; Intron, Gyeonggi-do, Korea), both forward and reverse primers (0.4 mM) and template (*ca.* 50–150 ng of DNA). Thermocycling conditions were the same as for real-time PCR. Amplification products were purified (AxyPrep PCR cleanup kits, Axygen, Union City, CA, USA) and sequenced in both directions using the primers from real-time PCR assay by an external contractor (University of Waikato DNA Sequencing Facility, Hamilton, New Zealand). The resulting sequences were compared to existing sequences in GenBank using the BLAST online software.

## 4. Conclusions

At present real-time PCR is the most cost-effective, sensitive, and rapid molecular technique for the detection and quantification of dinoflagellates from environmental samples [62]. The assays developed in this study demonstrate great potential for aiding in monitoring programmes for both food safety purposes and rapid screening of samples for species of interest. These assays are all utilised regularly as part of the New Zealand Marine Phytoplankton Monitoring Programme [24], as support for LM analyses by confirming the identification of toxic species in water samples. However, as multiplexing techniques, sequencing technologies, genomic and bioinformatic resources all improve, it is likely that molecular techniques will be increasingly utilised for routine phytoplankton monitoring programmes [34,45,62].
